# Impact of waterpipe tobacco taxation on consumption, government revenue and premature deaths averted in Jordan, Lebanon and Palestine: a simulation study

**DOI:** 10.1136/tc-2022-057284

**Published:** 2022-12-13

**Authors:** Mohammed Jawad, Sameera Awawda, Ruba Abla, Ali Chalak, Yousef S Khader, Rima T Nakkash, Aya Mostafa, Ramzi G Salloum, Niveen M E Abu-Rmeileh

**Affiliations:** 1 Public Health Policy Evaluation Unit, Imperial College London, London, UK; 2 Department of Economics and Institute of Community and Public Health, Birzeit University, Birzeit, Palestine; 3 Department of Health Promotion and Community Health, American University of Beirut, Beirut, Lebanon; 4 Department of Agriculture, American University of Beirut, Beirut, Lebanon; 5 Epidemiology and Biostatistics, Jordan University of Science and Technology, Irbid, Jordan; 6 Health Behaviour and Education Department, American University of Beirut, Beirut, Lebanon; 7 Department of Community, Environmental, and Occupational Medicine, Faculty of Medicine, Ain Shams University, Cairo, Egypt; 8 Health Outcomes and Policy, University of Florida, Gainesville, Florida, USA; 9 Institute of Community and Public Health, Birzeit University, Ramallah, Palestine

**Keywords:** Taxation, Economics, Global health, Harm Reduction, Low/Middle income country

## Abstract

**Introduction:**

Despite the high prevalence of waterpipe tobacco smoking in the Eastern Mediterranean region, evidence supporting its fiscal measures is limited. We modelled the impact of waterpipe tobacco-specific excise taxes on consumption, government revenue and premature deaths averted in Jordan, Lebanon and Palestine.

**Methods:**

We developed a simulation model using country-specific and market share-specific price, consumption and price elasticity data from WHO, UN Comtrade and nationally representative surveys. We modelled increases to specific excise taxes to meet a 35.9% tax burden on 20 g of waterpipe tobacco in Lebanon and Jordan, in line with the global average, and to double government revenues from excise duties in Palestine, which has surpassed this average.

**Results:**

Specific excise tax was raised by $1.14 ($0.18–$1.32) in Jordan, $2.41 ($0.03–$2.44) in Lebanon (alongside removal of *ad valorem* taxes) and $2.39 ($1.72–$4.11) in Palestine per 20 g of waterpipe tobacco. Government revenue increased by $126.3 million in Jordan, $53.8 million in Lebanon and $162.4 million in Palestine while waterpipes smoked decreased by 32.4% in Jordan, 71.0% in Lebanon and 16.3% in Palestine. The corresponding numbers of premature deaths averted annually were approximately 162 000; 1 000 000; and 52 000.

**Discussion:**

Increases in waterpipe tobacco-specific excise taxes substantially reduce smoking and increase government revenue and averted premature deaths in Jordan, Lebanon and Palestine. This has positive implications for both public health and financing and should be considered a policy priority.

**WHAT IS ALREADY KNOWN ON THIS TOPIC**

- Waterpipe tobacco tax rates are lower than cigarette tax rates in most countries.
- Evidence supporting waterpipe tobacco taxation is severely lacking.

**WHAT THIS STUDY ADDS**

- Waterpipe tobacco taxation is likely to be an effective policy measure.
- Substantial fiscal and public health gains result from an increase to the specific excise tax component of waterpipe tobacco.

**HOW THIS STUDY MIGHT AFFECT RESEARCH, PRACTICE OR POLICY**

- This study contributes to understanding of how waterpipe tobacco taxation impacts fiscal or public health outcomes.
- This study presents evidence to inform how waterpipe taxation policies may affect waterpipe tobacco market shares.

## Introduction

Taxing tobacco products is considered one of the most effective policies to improve government revenue and protect public health. WHO considers total taxes amounting to at least 75% of the retail price of tobacco products (‘the tax burden’) as best practice.^1^ In 2020, forty countries were protected by tax rates at 75% or more of the price of the most popular brand of cigarettes, including several countries in the Eastern Mediterranean region such as Egypt (78.5%), Jordan (78.0%), Morocco (76.1%) and Palestine (92.8%).^1^ However, equally strong taxation policies are lacking for waterpipe tobacco. For example, only 14 of 22 countries in the Eastern Mediterranean report having taxes for waterpipe tobacco, of whom just four (Iran, Lebanon, Libya and Oman) have rates equal to or higher than cigarette tax rates.^2^ In 2019, the average tax burden for waterpipe tobacco from 13 countries was 35.9%^1^; however, it is not clear whether this is calculated for the most popular brand or averaged across the entire market, nor whether the tax burden reflects home or café smoking. This is important because smoking waterpipe tobacco in a café comes with an extremely high industry mark-up in price to account for the café service, and so the tax burden on café smoking is likely to be extremely low and therefore ineffective to prevent smoking.

Tobacco tax simulation models including SimSmoke, the WHO Tax Simulation Model and the Tobacco Excise Tax Simulation (TETSiM) model have been applied to many countries worldwide, including in the Caribbean,^3^ China,^4^ West Africa^5^ and high-income settings.^6 7^ These models provide evidence on the potential impact of tax increases on government revenue, smoking cessation and public health outcomes, and provide a powerful tool with which to engage policy makers and empower public health advocates. However, the limited number of studies that have simulated tax models in the Eastern Mediterranean region assesses cigarettes only.^8 9^ This is of concern as the Eastern Mediterranean region is projected to make the least progress globally in reducing its rates of smoking and has low adaptation of MPOWER policies.^10^

The attention deficit to waterpipe tax policy may in part be due to the limited available evidence, the absence of which is particularly striking given high prevalence estimates of waterpipe tobacco in many Eastern Mediterranean countries.^11^ For example, recent national surveys have estimated current waterpipe tobacco use as 11.0% in Jordan, 39.5% in Lebanon and 12.9% in the West Bank (Palestine).^12^ A recent systematic review found only one study, conducted in Lebanon in 2013, that measured the price elasticity of demand for waterpipe tobacco smoking.^13^ Our research group has since produced cross-national estimates of waterpipe tobacco price elasticities in Jordan, Lebanon and the West Bank,^14^ though these elasticities have yet to be used in models that simulate demand, government revenue and public health outcomes under different waterpipe tax scenarios.

The Eastern Mediterranean region urgently requires research on the economics of waterpipe tobacco to inform policy debates. To the best of our knowledge, no prior research has simulated waterpipe taxation in any context. Therefore, the aim of this paper is to model the impact of increasing waterpipe tobacco taxes on demand, government revenue and premature deaths averted in Jordan, Lebanon and Palestine.

## Methods

We developed a simulation model that used country-specific and market share-specific price data, consumption data and price elasticities of demand to calculate the change in consumption, government revenue and premature deaths averted following an increase in the specific excise tax of waterpipe tobacco. We adapted our model from TETSiM^15^ and programmed it in Microsoft Excel. This study was exempted from ethical approval as no human subjects were involved.

### Data

We used country-level waterpipe tobacco tax structures from the 2021 WHO Report on the Global Tobacco Epidemic.^1^ The WHO report compiled these data in 2020 and reported the unit of waterpipe tobacco as 20 g, which is the approximate weight of tobacco used in one waterpipe smoking session. WHO regional data collectors gather taxation data directly from ministries of finance and check their validity against other sources such as tax law documents and official schedules. The WHO report focuses on indirect taxes only (eg, excise tax, import duties and value-added taxes (VAT)) and does not consider direct taxes such as corporate taxes due to the practical difficulty in obtaining information on them and the complexity in estimating their potential impact on price in a consistent manner.

We obtained country-level import prices, also known as costs, insurance and freight (CIF) prices, from UN Comtrade, the largest depository of international trade data.^16^ UN Comtrade has a specific category for waterpipe tobacco (code 240311), and reports import and export weights and prices for each country. The value of these trades is then converted from national currency into US dollars using exchange rates supplied by the reporter country. We calculated the CIF as the value per 20 g of waterpipe tobacco and used data from 2019 for each county. In the absence of available data, we assumed the CIF price was identical to the ex-factory price for domestically produced waterpipe tobacco, and tested this assumption in a sensitivity analysis (see below).

We derived market share-specific waterpipe price data, consumption data and price elasticities of demand directly from nationally representative tobacco use surveys we conducted in 2019, which asked individuals the price, quantity and location of their last waterpipe tobacco purchase, in addition to a volumetric choice experiment that yielded estimates of price elasticities of demand.^14^ We used point elasticities estimated using zero-inflated count models that assumed a constant price elasticity of demand, the details of which are reported elsewhere.^14^ We calculated market shares based on the self-reported expenditure on waterpipe tobacco in these surveys. Market shares were first stratified by location of smoking because prices vary substantially depending on whether waterpipe tobacco is smoked at home or in a café. Once stratified by location, the median price at last purchase was used to categorise respondents into two groups: discount (below the median) and premium (above the median). The four market shares were (1) discount home use, (2) premium home use, (3) discount café use and (4) premium café use.

For each market share, we calculated the country-level annual number of waterpipes smoked. This was done by multiplying the mean number of waterpipe sessions per day, the mean number of heads (ie, servings of waterpipe tobacco) per session (assuming 20 g per head), the prevalence of current waterpipe tobacco (all taken from our tobacco use household surveys) and the adult population over 15 years (taken from the World Bank^17^). As our consumption data were taken from household surveys that would have captured both legal and illicit consumption, we downscaled our consumption estimates to account for illicit use and to avoid overestimating government revenue. We estimated illicit trade at 7% in Jordan,^18^ 25.9% in Lebanon^18^ and 25.0% in Palestine.^19^ By having market share-specific prices, quantities consumed and tax structures, we were able to calculate market-weighted tax burdens which were substantially below those reported by WHO (17.5% vs 39.8% for Jordan; 17.4% vs 25.4% for Lebanon; 44.4% vs 79.0% for Palestine).^1^

### Model

Data inputs for the model are found in online supplemental table S1. The model followed a structure similar to previously published models in this field.^5 15^ We decomposed the retail price of 20 g of waterpipe tobacco into six components: the CIF/ex-factory price, import duty, specific tax, *ad valorem* tax, VAT and industry margin. We then applied country-specific and market share-specific waterpipe tobacco elasticity estimates^14^ to calculate the new annual demand (number of waterpipes smoked), new government revenues (total received from import, excise and VAT) and premature deaths averted.

10.1136/tc-2022-057284.supp1 Supplementary data

We used the following formula to estimate new consumption values following a change in underlying tax rates from baseline (period 0) to follow-up (period 1):

$$Q_{1im}=Q_{0im}{\left(P_{1im}/P_{0im}\right)}^{\varepsilon_{im}}$$

where *i* is the country, *m* is the market share*, Q* is the quantity of 20 g waterpipe tobacco units consumed, *ε* is the own-price elasticity of demand and *P* is the retail price. This formula avoids overestimating demand responses where tax increases are large, by basing our simulations on an algebraically ‘exact’ (ie, exponential decay) rather than ‘approximate’ (ie, linear decrease) relationship between per cent price and demand changes that is captured by price elasticities. We calculated premature deaths by using a cigarette modelling assumption that half of the reduction in waterpipes smoked was due to people quitting (rather than cutting down), of whom 35% would have died prematurely had they not quit.^15^ We felt it was acceptable to apply this assumption to waterpipe tobacco based on a meta-analysis showing a similar harm profile of both products.^20^

Lebanon was the only country of the three to have an import tax on waterpipe tobacco, and our national survey estimated that the entire Lebanese waterpipe market is made up of imported products. We therefore applied the import tax to all market shares in Lebanon. In the absence of available tax bases from policy documents and following previous assumptions made in the field,^5^ we assumed that import duty was applied to the CIF price, that the specific excise was applied to each 20 g of waterpipe tobacco, that the *ad valorem* excise was applied to the sum of CIF and import duty and that the VAT was applied to the sum of CIF, import duty, specific excise, *ad valorem* excise and industry margin. The industry margin was calculated as the retail price minus the sum of all taxes and the CIF price. In modelling tax scenarios, we assumed no changes in the prevalence of illicit trade, CIF price or industry margin (ie, we assumed production costs remained constant and the industry passed the whole cost of the tax onto the consumer without overshifting or undershifting their prices in response,^21^ the latter two of which were tested in a sensitivity analysis) (see below).

We calibrated our models to match the publicly available tobacco tax revenues from each country and ensure our simulations were within the expectations of policy makers. We used data from the WHO Global Health Observatory which reported annual tax revenues from specific and *ad valorem* tobacco taxes at $996 million from all tobacco products in Jordan in 2019; $64 million from all tobacco products in Lebanon in 2019; and $278 million from cigarettes only in Palestine in 2015 (adjusted to $282 million in 2019).^22^

### Analysis

We presented the base scenario for each country, reflecting current demand and government revenue based on existing waterpipe tobacco tax structures. We then presented the demand, government revenue, price structure and premature deaths averted under a new scenario for each country. For Jordan, we modelled a specific excise tax increase of $1.14 (from $0.18 to $1.32) per 20 g, leaving other tax rates unchanged, to meet a market-weighted tax burden of 35.9%, matching the WHO-reported mean tax rate for waterpipe tobacco products globally. Similarly for Lebanon, we modelled a specific excise tax increase of $2.01 (from $0.03 to $2.04) per 20 g to meet the 35.9% tax burden, but additionally removed their *ad valorem* excise tax to simplify the overall structure. As Palestine’s current tax burden was already above 35.9% (at 44.4%), we modelled a specific excise tax increase of $2.39 (from $1.72 to $4.11) per 20 g that would double the government revenue from excise taxes.

### Sensitivity analysis

We performed sensitivity analyses to address the underlying uncertainties or potentially highly influential parameters. The first uncertainty was our assumption that the CIF (import) price was the same as the ex-factory price (domestic cost of production), so we tested new CIF prices that were 50% higher or lower than the original CIF price. The second uncertainty was our assumption that the industry would not respond to a change in taxation by undershifting or overshifting their pricing strategy. We therefore included a 10% industry overshift or undershift. Finally, we altered our market share-specific elasticity estimates to the lower and upper 95% CIs of the original estimate (as reported in Chalak *et al* 14), as these were likely to be the main influencing parameters on our results. We report the results of our sensitivity analyses on consumption, government revenue and premature deaths averted, expressed as a relative percentage difference from the main scenario analysis.

## Results

Tax structures for each country’s base and new scenario are presented in online supplemental table S2.

### Jordan

In the base scenario, we estimated that the market-weighted average retail price for 20 g of waterpipe was $3.43, ranging from $0.64 in discount home to $7.38 in premium café market shares. About three-quarters of the 168.0 million waterpipes smoked in Jordan each year were in premium market shares (37.7% in premium cafés, 36.7% in premium home). The market-weighted tax burden was 17.5%, ranging from 2.5% in premium cafés to 28.7% in discount home market shares. Most of the Jordanian government’s $100.5 million revenue from waterpipe tobacco came from VAT ($69.7 million) rather than excise taxes ($30.8 million).

**Figure 1:**
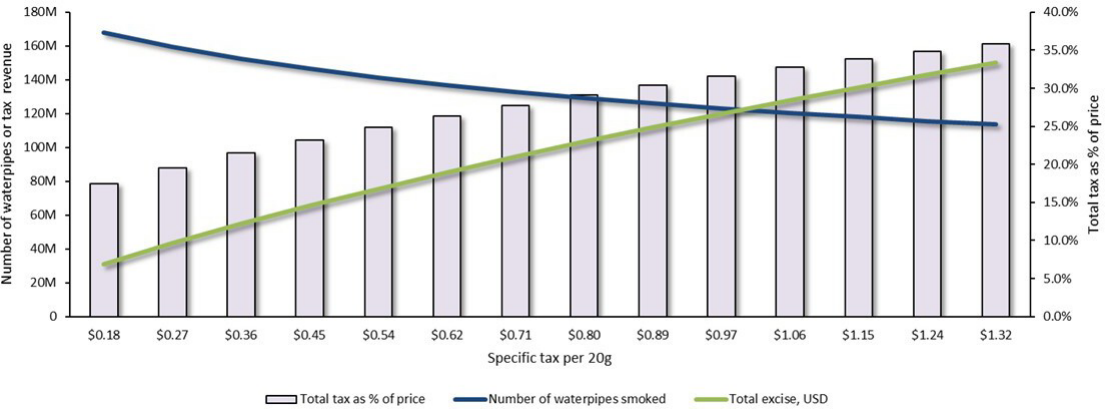
The impact of waterpipe tobacco tax increases on the annual number of waterpipes smoked, government revenue from excise and market-weighted tax burden as a percentage of retail price in Jordan.

Raising the market-weighted tax burden from 17.5% to 35.9% required a specific excise tax increase by $1.14, from $0.18 to $1.32 per 20 g of waterpipe tobacco, all else being equal. The effects of an incremental increase in the specific tax on excise revenue and consumer demand to meet this target are shown in figure 1. Such an increase would raise the average retail price by $2.15 (62.7%), from $3.42 to $5.57 per 20 g, and raise the total annual tax revenue by $126.3 million (125.6%), from $100.6 million to $226.9 million, of which $119.3 million (94.5%) would come from the specific tax. This tax increase would reduce the number of waterpipes smoked annually by 54.4 million (32.4%), from 168.0 to 113.6 million, while averting approximately 162 000 premature deaths.

### Lebanon

The base scenario estimated a market-weighted average retail price for 20 g of waterpipe at $3.82, ranging from $0.64 in discount home to $7.92 in premium café market shares. About 70.0% of the 198.5 million waterpipes smoked in Lebanon each year were in premium market shares (41.4% in premium cafés, 28.5% in premium home). The market-weighted tax burden was 17.3%, ranging from 5.3% in premium cafés to 34.9% in discount home market shares. Just under half of the Lebanese government’s $131.2 million revenue from waterpipe tobacco came from excise ($61.9 million).

**Figure 2:**
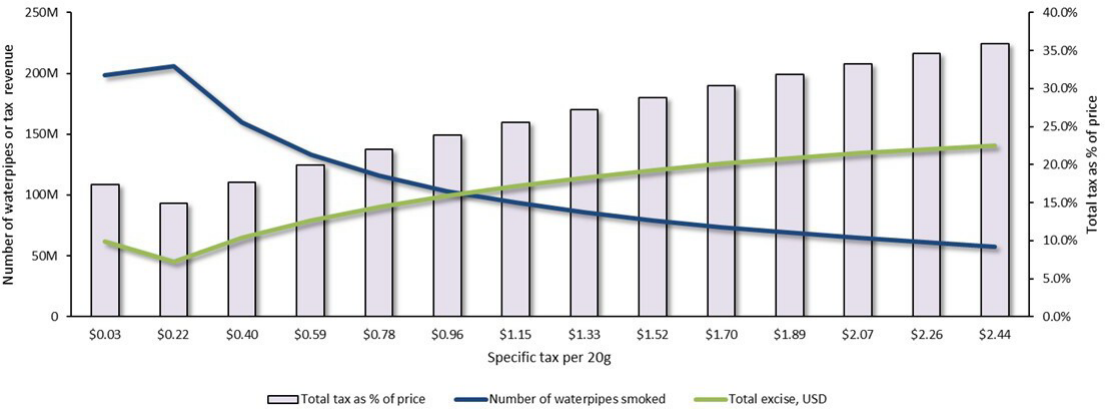
The impact of waterpipe tobacco tax increases on the annual number of waterpipes smoked, government revenue from excise and market-weighted tax burden as a percentage of retail price in Lebanon.

Raising the market-weighted tax burden from 17.3% to 35.9% while removing the *ad valorem* excise tax required a specific excise tax increase by $2.41, from $0.03 to $2.44 per 20 g of waterpipe tobacco, all else being equal. The effects of an incremental increase in the specific tax on excise revenue and consumer demand to meet this target are shown in figure 2. It shows that a specific excise of $0.34 compensated for the lost excise caused by removing the *ad valorem* tax. The increase in specific excise to $2.44 would raise the average retail price by $5.13 (134.5%), from $3.81 to $8.95 per 20 g, and raise the total annual tax revenue by $53.8 million (37.0%), from $131.2 to $184.9 million, of which $140.6 million (76.0%) would come from the specific tax. This tax increase would reduce the number of waterpipes smoked annually by 140.9 million (71.0%), from 198.5 to 57.6 million, while averting approximately 1 000 000 premature deaths.

### Palestine

In the base scenario, we estimated a market-weighted average retail price for 20 g of waterpipe at $5.34, ranging from $2.40 in discount market shares to $7.62 in premium café shares. Nearly half (48.1%) of the 68.6 million waterpipes smoked in Palestine each year were smoked in discount café market shares. The market-weighted tax burden was 44.4%, ranging from 22.6% in premium cafés to 71.7% in home market shares. Just under three-quarters of the Palestinian government’s $162.4 million revenue from waterpipe tobacco came from excise taxes ($118.1 million).

**Figure 3:**
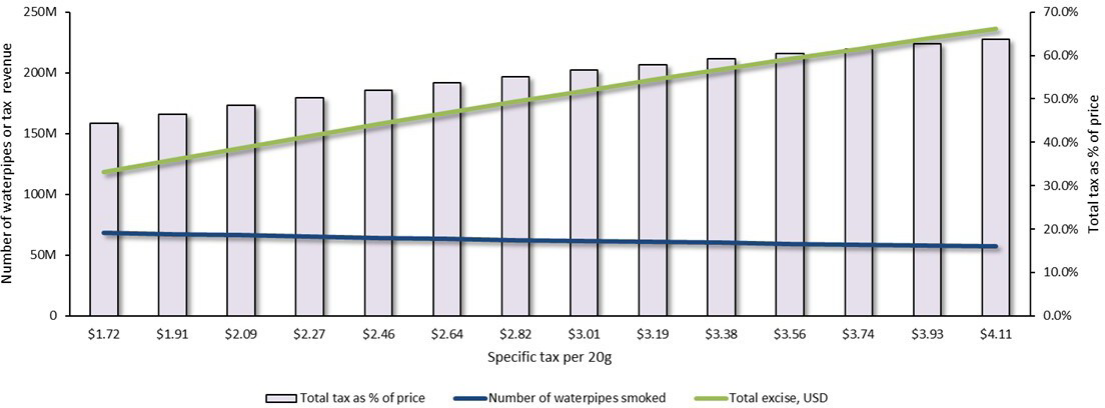
The impact of waterpipe tobacco tax increases on the annual number of waterpipes smoked, government revenue from excise and market-weighted tax burden as a percentage of retail price in Palestine.

A doubling of revenue from excise taxes required a specific excise tax increase by $2.39, from $1.72 to $4.11 per 20 g of waterpipe tobacco, all else being equal. The effects of an incremental increase in the specific tax on excise revenue and consumer demand to meet this target are shown in figure 3. Such an increase would raise the average retail price by $2.15 (54.9%), from $3.42 to $5.57 per 20 g, and raise the market-weighted tax burden by 19.3%, from 44.4% to 63.7%. This tax increase would reduce the number of waterpipes smoked annually by 11.2 million (16.3%), from 68.6 to 57.4 million, while averting approximately 52 000 premature deaths.

### Sensitivity analysis

Our results remained robust to sensitivity analyses in terms of direction and magnitude of effect estimates (online supplemental table S3). Increasing or decreasing the CIF value by 50% had a negligible effect on waterpipe consumption, government revenue and premature deaths averted, changing our results by 0.6%–1.4% in Jordan, 1.6%–3.6% in Lebanon and 0.3%–1.4% in Palestine depending on the outcome. Introducing a 10% industry overshift or undershift affected the model outcomes by 1.5%–6.1% in Jordan, 5.2%–16.4% in Lebanon and 1.2%–3.5% in Palestine depending on the outcome.

Our simulations proved robust to changes in the values of elasticity estimates, whereby setting these to their upper and lower 95% CIs had relatively small effects on outcomes in all three countries. In Jordan, premium home market share elasticities had the largest effects on outcomes compared with other market share elasticities, altering our results by 3.2%–6.3% depending on the outcome. In Lebanon, revised elasticity estimates in all market shares altered our results by below 2.5% and more often by less than 1%. In Palestine, discount café market share elasticities had the largest effects on outcomes compared with other market share elasticities, altering our results between 0.4% and 5.5% depending on the outcome. In summary, except for the large effect of industry undershifting or overshifting in Lebanon on consumption and government revenue (changing model results by 11.9%–16.4%), all other sensitivity analyses altered our results by less than 6.3%.

## Discussion

Our waterpipe tobacco model estimated that increases in the specific excise tax can yield substantial government revenue and public health gains in Jordan, Lebanon and Palestine. Raising the specific tax in Jordan by $1.14 (from $0.18 to $1.32) per 20 g of waterpipe tobacco would increase government revenue by 125.6%, reduce consumption by 32.4% and avert 162 000 premature deaths. Raising the specific tax in Lebanon by $2.41 (from $0.03 to $2.44) and removing the *ad valorem* component would increase government revenue by 37.0%, reduce consumption by 71.0% and avert 1 000 000 premature deaths. Raising the specific tax in Palestine by $2.39 (from $1.72 to $4.11) would double the government revenue from excise duties, reduce consumption by 16.3% and avert 52 000 premature deaths. It is imperative that policy makers are made aware of the government revenue potential and public health gains that can be attained by a logistically simple alteration in tax rates.

Our study has highlighted the importance of using market-weighted tax burdens when simulating tax increases in waterpipe tobacco policy. It is not clear whether the WHO Global Report on the Tobacco Epidemic reports waterpipe tax structures and retail prices for a single waterpipe brand and ignores the tax to price ratio at waterpipe cafés where a substantial industry margin (at least 60%) and consumer base are found (waterpipe was last smoked in a café among 19.3% of smokers in Jordan, 33.9% in Lebanon and 72.9% in Palestine). Our base scenarios estimated that taxes constituted 17.5%, 17.3% and 44.4% of the market-weighted waterpipe tobacco retail price in Jordan, Lebanon and Palestine, respectively, whereas WHO reports these as 39.8%, 25.4% and 79.0%,^1^ which resemble our model’s estimate for home market shares only. Our model may have therefore brought to light a systemic tendency by WHO to underestimate the impact of waterpipe tobacco taxation in the Eastern Mediterranean countries under study, and this must be addressed in future WHO reports.

This study has also shown the importance of national contexts in assessing the differential effects of taxation in each country. Jordan and Lebanon, for example, have similarly structured waterpipe tobacco market share sizes, prices and tax rates, but their different price elasticity estimates result in Lebanon seeing less consumption and Jordan seeing more government revenue at the 35.9% tax burden. In Palestine, which has very inelastic price elasticities compared with Jordan and Lebanon, increasing specific taxes can result in large government revenue gains without reducing consumption as considerably. Different national contexts require tailored dialogue with policy makers instead of a one-size-fits-all approach.

The core strengths of this paper include the disaggregation of prices, quantities consumed, price elasticities and location of use by country and market share. Our tobacco surveys employed random sampling and were nationally representative, minimising the possibility of sampling error bias in our models. This study shows the excellent potential of high-quality surveillance data to inform tobacco control policy and advocacy objectives, and variables used in this model should be incorporated into routine tobacco surveys. Our results also remained robust to sensitivity analyses. We used a well-known and peer-reviewed model structure that manipulates only specific excise taxes and provides easily understandable results that will facilitate discussion with policy makers. Our inclusion of premature deaths averted further supports the public health case for increased taxation on waterpipe tobacco products.

However, our model is not without its limitations. Its cross-sectional design restricts us from making short-term and long-term tax projections on demand, government revenue and premature deaths averted, although the data provided here could be used in future statistical forecasts or dynamic Markov models. Indirect fiscal benefits from quitting, such as fewer hospital admissions attributed to smoking, were not incorporated into the model and hence our revenue estimates are conservative. The model also did not incorporate cross-price elasticities of demand, and it is possible that waterpipe tobacco smokers may find substitute products (including moving to other waterpipe tobacco brands within a market share or between market shares altogether) in the face of rising prices. However, it is important that policy makers raise taxes of all tobacco products simultaneously to discourage product substitution, and our previous work in this field showed inconsistent or no substitution effects between café and home market shares.^14^ Another limitation is that our assumptions about the behaviour of consumers and the industry may not hold in the face of large tax increases, so it remains important to measure consumer demand and industry behaviour in response to tax increases in Jordan, Lebanon and Palestine to feed back into future iterations of our model. We have, however, avoided inflated demand change estimates by using exact rather than approximate relationships between taxation and demand.

In conclusion, increases to the specific excise tax of waterpipe tobacco in Jordan, Lebanon and Palestine are likely to yield substantial government revenue and improve public health. We hope that this paper provides a clear and informative model for policy makers to improve government financing while protecting public health. Future research should consider the impact of waterpipe tobacco taxation on sociodemographic inequalities, and ensure evaluative measures are in place for future changes to waterpipe tobacco taxes.

## Data Availability

Data are available in a public, open access repository.
