# Supplementary material for: Impact of waterpipe tobacco taxation on consumption, government revenue and premature deaths averted in Jordan, Lebanon and Palestine: a simulation study
Source: Tob Control. 2022 Dec 13;33(e1):e85–90. doi: 10.1136/tc-2022-057284 (PMC10958304; doi:10.1136/tc-2022-057284)
Supplement: Supplementary data [file tc-2022-057284supp001.pdf]

Supplementary Tables

Table S1. Waterpipe tobacco data inputs

**Variable**	**Input value**			**Source**
	**Jordan**	**Lebanon**	**Palestine**	
Import duty tax, % of CIF/ex-factory price	0.0	35.1	0.00	WHO 2021 [1]
Specific excise tax per 20g, USD	0.18	0.03	1.72	WHO 2021 [1]
Value added tax, % of retail price	13.8	8.6	13.8	WHO 2021 [1]
Annual imports, trade value, USD	4,447,191	28,076,527	808,859	UN Comtrade 2019 [2]
Annual imports, netweight, kg	909,208	3,042,453	79,760	UN Comtrade 2019 [2]
CIF/ex-factory price per 20g, USD	0.10	0.13	0.20	Annual imports trade value/(netweight*1000/20)
Discount café share, %	3.9	4.3	48.1	Chalak *et al*., 2021 [3]
Premium café share, %	37.7	41.4	24.4	Chalak *et al*., 2021 [3]
Discount home share, %	21.8	25.8	10.7	Chalak *et al*., 2021 [3]
Premium home share, %	36.7	28.5	16.8	Chalak *et al*., 2021 [3]
Discount café retail price, USD	3.37	3.50	5.86	Chalak *et al*., 2021 [3]
Premium café retail price, USD	7.38	7.92	7.62	Chalak *et al*., 2021 [3]
Discount home retail price, USD	0.64	0.64	0.73	Chalak *et al*., 2021 [3]
Premium home retail price, USD	1.02	0.78	2.40	Chalak *et al*., 2021 [3]
Discount café own price elasticity	-0.335	-1.699	-0.291	Chalak *et al*., 2021 [3]
Premium café own price elasticity	-0.674	-2.312	-1.120	Chalak *et al*., 2021 [3]
Discount home own price elasticity	-0.915	-1.700	-0.650	Chalak *et al*., 2021 [3]
Premium home own price elasticity	-0.601	-1.949	0.196*	Chalak *et al*., 2021 [3]
Current use, %	10.9	39.5	12.9	Chalak *et al*., 2021 [3]
Illicit (non-tax paid) consumption, % of current use	7.0	25.9	25.0	Online reports [4,5]
Population size aged >15 years	6,851,000	5,114,372	2,960,687	World Bank [6]
Number of annual 20g sessions	361,190,280	1,339,392,106	217,858,689	Current use * Population size * Mean number of waterpipes/day [3] * Mean number of heads/waterpipe [3] * 365.25

CIF: Cost, insurance, and freight; *set to 0 following peer review

Table S2. Tax structures per 20g and market-weighted retail price of 20g of waterpipe tobacco for each simulated scenario, USD

Country	Scenario	CIF price	Import duty	Specific excise	*Ad valorem* excise	VAT	Margin	Retail price
**Jordan**	Base	0.10	0.00	0.18	0.00	0.42	2.73	3.43
	Tax is 35.9% of retail price	0.10	0.00	1.32	0.00	0.68	3.48	5.57
**Lebanon**	Base	0.13	0.00	0.03	0.28	0.30	3.02	3.82
	Tax is 35.9% of retail price	0.13	0.06	2.04	0.00	0.66	5.44	8.38
**Palestine**	Base	0.20	0.00	1.72	0.00	0.65	2.77	5.34
	Double government revenue from excise	0.20	0.00	4.11	0.00	0.97	2.70	7.98

CIF: costs, insurance, and freight; VAT: Value Added Tax;

Table S3. Sensitivity analysis showing the impact of different parameter changes on consumption (C), government revenue from all taxes (G), and premature deaths averted (P), expressed as a percentage relative to the new scenario.

**Variable**	**Jordan**			**Lebanon**			**Palestine**		
	C	G	P	C	G	P	C	G	P
Cost, insurance, and freight price or ex-factory price +50%	-1.0	-0.6	+1.4	-3.4	-2.0	+1.6	-0.6	-0.3	+1.4
Cost, insurance, and freight price or ex-factory price -50%	+1.0	+0.6	-1.4	+3.6	+2.0	-1.7	+0.6	+0.3	-1.4
Discount café own price elasticity lower 95% confidence interval	-0.4	-0.4	+0.1	-0.3	-0.3	+0.1	-4.8	-4.9	-0.4
Discount café own price elasticity upper 95% confidence interval	+0.5	+0.4	-0.1	+0.4	+0.3	-0.1	+5.4	+5.5	+0.4
Premium café own price elasticity lower 95% confidence interval	-2.4	-2.9	-1.4	-2.3	-2.4	-0.4	-1.7	-1.8	-0.5
Premium café own price elasticity upper 95% confidence interval	+2.5	+3.0	+1.4	+2.3	+2.4	+0.3	+1.8	+2.0	+0.5
Discount home own price elasticity lower 95% confidence interval	-2.9	-2.2	+1.9	-0.9	-0.8	+0.6	-2.7	-2.5	+1.0
Discount home own price elasticity upper 95% confidence interval	+3.8	+3.0	-2.4	+1.1	+0.9	-0.7	+4.1	+3.8	-1.4
Premium home own price elasticity lower 95% confidence interval	-5.2	-4.2	+3.2	-0.9	-0.7	+0.6	N/A	N/A	N/A
Premium home own price elasticity upper 95% confidence interval	+6.3	+5.0	-3.4	+1.0	+0.8	-0.7	N/A	N/A	N/A
Industry over-shift 10%	-3.4	-1.5	+6.0	-12.9	-11.9	+5.2	-1.8	-1.2	+3.4
Industry under-shift 10%	+3.9	+1.7	-6.1	+16.4	+14.9	-5.6	+2.0	+1.3	-3.5

N/A: Not applicable as elasticity artificially set to 0 following peer review

References

1. World Health Organization. WHO report on the global tobacco epidemic 2021: addressing new and emerging products [online]. Available at: https://bit.ly/31slvCZ [Accessed 05 November 2021].
2. United Nations. UN Comtrade Database [online]. Available at: https://bit.ly/3GGaQVF [Accessed 28 October 2021].
3. Chalak A, et al. Own-price and cross-price elasticities of demand for cigarettes and waterpipe tobacco in three Eastern Mediterranean countries: a volumetric choice experiment. Tobacco control. 2021. Epub 2021/07/02. doi: 10.1136/tobaccocontrol-2021-056616
4. Oxford Economics. Levant Illicit Tobacco 2019 [online]. Available at: https://bit.ly/3dBmXt0 [Accessed 17.08.22].
5. Philip Morris International. Israel's COVID-19 measures have a promising impact on illicit cigarette trade [online]. Available at: https://bit.ly/3dpKvkf [Accessed 17.08.22].
6. World Bank. Data. Population, total [online]. Available at: https://bit.ly/3pL8n61 [Accessed 28 October 2021].
